# Supplementary material for: Spatial dealiasing of classical geomagnetic survey data through use of a microfabricated wearable quantum magnetometer
Source: Sci Rep. 2026 May 29;16:24581. doi: 10.1038/s41598-026-54706-7 (PMC13454324; doi:10.1038/s41598-026-54706-7)
Supplement: Supplementary file 1 [file 41598_2026_54706_MOESM1_ESM.pdf]

Spatial dealiasing of classical geomagnetic survey data through use of a microfabricated wearable quantum magnetometer - Supplementary

Stirling Scholes[1,*], Alissa Forsythe[2], Courtney Dyer[1], Amy Gilligan[3], Karen Lythgoe[2], Jenny Jenkins[4], Marcin Mrozowski[1], Jack-Andrew Smith[2], and Stuart Ingleby[1]

[1]Department of Physics, SUPA, University of Strathclyde, Glasgow G4 0NG, Scotland
[2]School of Geosciences, University of Edinburgh, Edinburgh, EH9 3FE, Scotland
[3]Department of Geology and Geophysics, University of Aberdeen, Aberdeen, AB24 3FX, Scotland
[4]Department of Earth Sciences, Durham University, Durham, DH1 3LE, England
[*]s.scholes@strath.ac.uk

Broad-scale modelling of the HBFZ

Simplified forward modelling of the PPM data was carried out to interpret the broad-scale magnetic signature of the full survey profile. Initial modelling focussed on reproducing the long-wavelength component of the anomaly, interpreted to originate from a large deeply-buried, strongly-magnetised source within the HBFZ. This feature was modelled as a rectangular prism approximately 3.7 km wide and with its upper surface at 1.6 km depth, similar to measurements from previous magnetic modelling at Blairgowrie[1] and constrained by the width of the mapped fault zone at this location. A vertically oriented body was initially adopted, as proposed by the previous model[1]. However, introducing a northward dip of $\sim$50° offers an improved fit to the observed data (see Figure 1) and is justified by surface observations of steep NW-dipping faults, along with evidence for lower-angle NW-dipping fault planes at depth[2–4]. The body was set to extend 5 km along strike, although this dimension was poorly constrained and greater lengths had minimal effect on the model response. Similarly, extension of the body below $\sim$15 km depth did not substantially change the calculated anomaly. Magnetic susceptibility was also varied, but ultimately fixed at 0.033 – a value higher than reported for serpentinite in the surrounding area[1], but within the recorded range for metamorphic or igneous material mapped within the fault zone.

After producing an acceptable fit to the long-wavelength component, the short-wavelength anomalies near the centre of the profile were modelled using three smaller, shallower bodies. These were interpreted to be igneous features (large dykes or basaltic andesite blocks within the fault zone) with a lower magnetic susceptibility of 0.020, also dipping northwards, and extending down to the top surface of the deeper body. The length along strike was smaller, set to 1 km. Other small residual anomalies along the profile were not modelled as they were attributed either to noise (e.g., in urban areas) or were not of interest to the overall architecture of the HBFZ (e.g., anomalies modelled in Figure 4). The final integrated model is shown in Figure 1, comprising a deep, northward-dipping body overlain by three shallower magnetic sources.

References

1. Farquharson, C. G. & Thompson, R. The Blairgowrie magnetic anomaly and its interpretation using simplex optimisation. *Earth Environ. Sci. Transactions The Royal Soc. Edinb.* **83**, 509–518, DOI: 10.1017/S026359330000585X (1992).

2. Hutton, D. H. W. Strike-slip terranes and a model for the evolution of the British and Irish Caledonides. *Geol. Mag.* **124**, 405–425, DOI: 10.1017/S0016756800017003 (1987).

3. Dentith, M. C., Trench, A. & Bluck, B. J. Geophysical constraints on the nature of the Highland Boundary Fault Zone in western Scotland. *Geol. Mag.* **129**, 411–419, DOI: 10.1017/S0016756800019506 (1992).

4. Ottemölller, L. & Thomas, C. Highland Boundary Fault zone: Tectonic implications of the Aberfoyle earthquake sequence of 2003. *Tectonophysics* **430**, 83–95, DOI: 10.1016/j.tecto.2006.11.002 (2007).

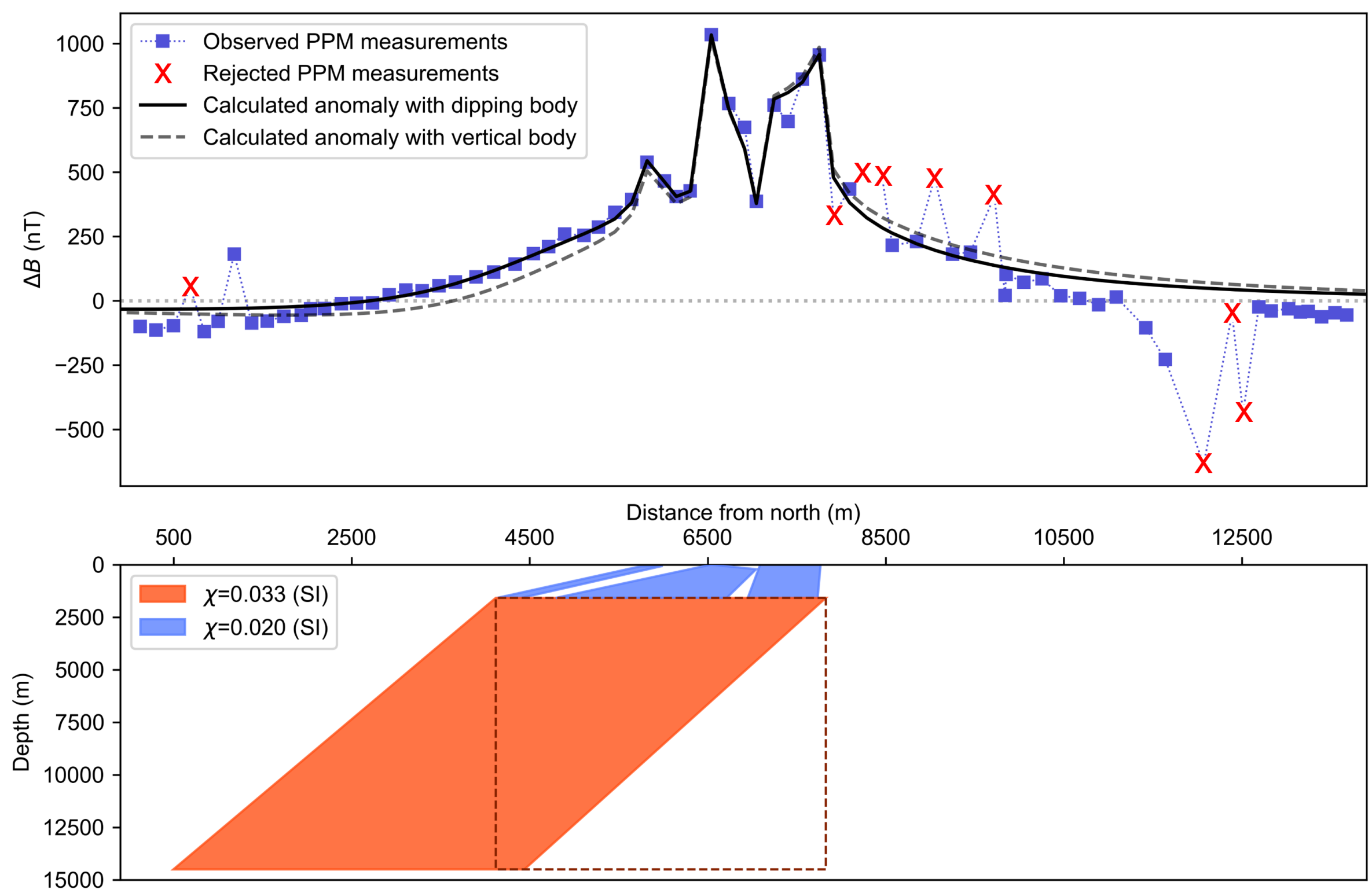

Figure 1. Forward modelling results for the full survey profile using the PPM data. The top panel shows the observed measurements projected onto the survey line (blue) and the calculated anomalies from two end-member models: one with a dipping body (solid black line) and one with a vertical body (dashed black line). Outliers in the PPM dataset have been removed based on high standard deviations in the OPM data. Note that the data projection is different from Figures 2-4 in the main manuscript for modelling purposes. The bottom panel shows the cross-sectional geometries of the corresponding modelled sources, including a large, deeply buried body (orange) with magnetic susceptibility $\chi = 0.033$ (SI units) and three smaller, shallow bodies (blue) with $\chi = 0.020$ (SI units).
